# Ag Nanoparticle–Functionalized Open-Ended Freestanding TiO_2_ Nanotube Arrays with a Scattering Layer for Improved Energy Conversion Efficiency in Dye-Sensitized Solar Cells

**DOI:** 10.3390/nano6060117

**Published:** 2016-06-15

**Authors:** Won-Yeop Rho, Myeung-Hwan Chun, Ho-Sub Kim, Hyung-Mo Kim, Jung Sang Suh, Bong-Hyun Jun

**Affiliations:** 1 Department of Bioscience and Biotechnology, Konkuk University, Seoul 143-701, Korea; rho72@snu.ac.kr (W.-Y.R.); hmkim0109@konkuk.ac.kr (H.-M.K.); 2 Department of Chemistry, Seoul National University, Seoul 151-747, Korea; hwanmc@hanmail.net (M.-H.C.); hosub@snu.ac.kr (H.-S.K.); jssuh@snu.ac.kr (J.S.S.)

**Keywords:** open-ended freestanding TiO_2_ nanotube arrays, dye-sensitized solar cells, plasmonic, scattering, anodization

## Abstract

Dye-sensitized solar cells (DSSCs) were fabricated using open-ended freestanding TiO_2_ nanotube arrays functionalized with Ag nanoparticles (NPs) in the channel to create a plasmonic effect, and then coated with large TiO_2_ NPs to create a scattering effect in order to improve energy conversion efficiency. Compared to closed-ended freestanding TiO_2_ nanotube array–based DSSCs without Ag or large TiO_2_ NPs, the energy conversion efficiency of closed-ended DSSCs improved by 9.21% (actual efficiency, from 5.86% to 6.40%) with Ag NPs, 6.48% (actual efficiency, from 5.86% to 6.24%) with TiO_2_ NPs, and 14.50% (actual efficiency, from 5.86% to 6.71%) with both Ag NPs and TiO_2_ NPs. By introducing Ag NPs and/or large TiO_2_ NPs to open-ended freestanding TiO_2_ nanotube array–based DSSCs, the energy conversion efficiency was improved by 9.15% (actual efficiency, from 6.12% to 6.68%) with Ag NPs and 8.17% (actual efficiency, from 6.12% to 6.62%) with TiO_2_ NPs, and by 15.20% (actual efficiency, from 6.12% to 7.05%) with both Ag NPs and TiO_2_ NPs. Moreover, compared to closed-ended freestanding TiO_2_ nanotube arrays, the energy conversion efficiency of open-ended freestanding TiO_2_ nanotube arrays increased from 6.71% to 7.05%. We demonstrate that each component—Ag NPs, TiO_2_ NPs, and open-ended freestanding TiO_2_ nanotube arrays—enhanced the energy conversion efficiency, and the use of a combination of all components in DSSCs resulted in the highest energy conversion efficiency.

## 1. Introduction

Since the original work by O’Regan and Grätzel in 1991 [1], dye-sensitized solar cells (DSSCs) have been investigated extensively because of their high energy conversion efficiency and low cost [2,3,4,5,6,7,8,9]. Generally, mesoporous TiO_2_ nanoparticle (NP) films and ruthenium sensitizers are used for DSSCs [2,3,4,10,11,12,13,14,15,16]. However, the efficiency of mesoporous TiO_2_ NP film–based DSSCs is limited by grain boundaries, defects, and numerous trapping sites. Moreover, mesoporous TiO_2_ NP films can cause charge recombination and mobility [17,18].

TiO_2_ nanotubes, which enhance electron transport and charge separation by creating direct pathways and accelerating charge transfer between interfaces, have great potential to overcome the problems of mesoporous TiO_2_ NP films [19,20,21,22]. TiO_2_ nanotubes can be prepared by a hydrothermal method [23] or an electrochemical method [24], known as anodization. TiO_2_ nanotube arrays prepared by anodization have a well-ordered and vertically oriented tubular structure that facilitates a high degree of electron transport and less charge recombination than mesoporous TiO_2_ NP films [25,26,27]. There is much room for improvement in the energy conversion efficiency of current DSSCs based on TiO_2_ nanotube arrays compared to the relatively extensively researched mesoporous TiO_2_ NP film–based DSSCs [28].

To date, several approaches for improving the energy conversion efficiency of TiO_2_ nanotube array–based DSSCs have been reported. Metal NPs, which can harvest light via surface plasmon resonance (SPR), have been used to enhance the energy conversion efficiency of DSSCs by introducing Au or Ag NPs into TiO_2_ nanotube arrays [29,30,31,32]. Barrier layers remove TiO_2_ nanotube arrays, so open-ended TiO_2_ nanotube arrays, which can also be classified as arrays of columnar nanopores, have been used for DSSCs to provide increased energy conversion efficiency [33]. Moreover, the energy conversion efficiency of TiO_2_ nanotube array–based DSSCs can be further increased by introducing a scattering layer to the active layer [34].

So far, TiO_2_ nanotubes that make use of a scattering layer [34] or plasmonic materials [14] have been reported, but a scattering layer with plasmonic materials has not been used in TiO_2_ nanotube–based DSSCs. In this study, we report the development of freestanding TiO_2_ nanotube arrays filled with Ag NPs and large TiO_2_ NPs, which improve the energy conversion efficiency of DSSCs. Furthermore, we compare the effects of Ag NPs and large TiO_2_ NPs in open- and closed-ended freestanding TiO_2_ nanotube arrays in DSSCs. The energy conversion efficiencies of the following eight types of DSSCs were compared: closed-ended freestanding TiO_2_ nanotube arrays with/without Ag NPs and/or a TiO_2_ scattering layer and open-ended freestanding TiO_2_ nanotube arrays with/without Ag NPs and/or a TiO_2_ scattering layer.

## 2. Results and Discussion

### 2.1. Structure of DSSCs with Freestanding TiO_2_ Nanotube Arrays with Channels Containing Ag NPs

**Figure 1:**
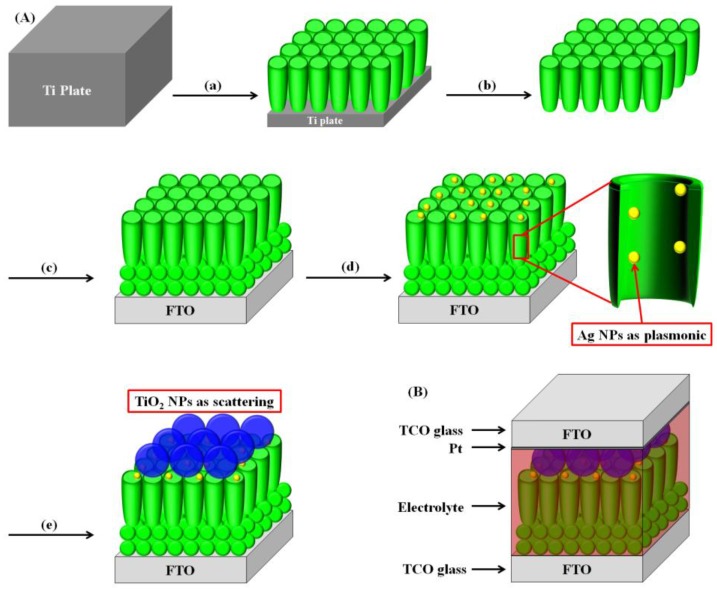
Overall scheme of dye-sensitized solar cells (DSSCs) with open-ended freestanding TiO_2_ nanotube arrays with Ag nanoparticles (NPs) and large TiO_2_ NPs. (**A**) (a) Ti anodization for TiO_2_ nanotube arrays; (b) freestanding TiO_2_ nanotube arrays and etching by ion milling; (c) transference of open-ended freestanding TiO_2_ nanotube arrays onto fluorine-doped tin oxide (FTO) glass; (d) formation of Ag NPs by ultraviolet (UV) irradiation; and (e) introduction of large TiO_2_ NPs. (**B**) Structure of a DSSC with freestanding TiO_2_ nanotube arrays and large TiO_2_ NPs.

Figure 1 illustrates the fabrication of DSSCs with Ag NPs and large TiO_2_ NPs to enable plasmonic and scattering effects in open-ended freestanding TiO_2_ nanotube array–based DSSCs. Ti plates were anodized and then annealed at 500 °C for 1 h to prepare anatase TiO_2_ nanotube arrays. After carrying out secondary anodization, the TiO_2_ nanotube arrays were easily detached from the Ti plates. TiO_2_ nanotube arrays, once separated from the Ti plates, are termed “closed-ended freestanding TiO_2_ nanotube arrays”. Freestanding TiO_2_ nanotube arrays have a barrier layer at the bottom that disturbs electron transport and electrolyte diffusion. This barrier layer was removed using the ion-milling method with several minutes of Ar^+^ bombardment to yield “open-ended freestanding TiO_2_ nanotube arrays”. The closed- and open-ended freestanding TiO_2_ nanotube arrays were transferred to fluorine-doped tin oxide (FTO) glass using TiO_2_ paste and annealed to enhance the adhesion between the closed- and open-ended freestanding TiO_2_ nanotube arrays and the fluorine-doped tin oxide (FTO) glass. To improve the energy conversion efficiency by the plasmonic effect, Ag NPs were embedded in the channel of freestanding TiO_2_ nanotube arrays using 254 nm ultraviolet (UV) irradiation with aqueous silver nitrate. To further enhance the energy conversion efficiency, large TiO_2_ NPs (400 nm) as a scattering layer were coated onto the active layer by the doctor blade method. This substrate was sandwiched with the counter electrode and filled with electrolyte. The active area of the DSSCs was ~0.25 cm^2^.

### 2.2. Characterization of Freestanding TiO_2_ Nanotube Arrays with Channels Containing Ag NPs

**Figure 2:**
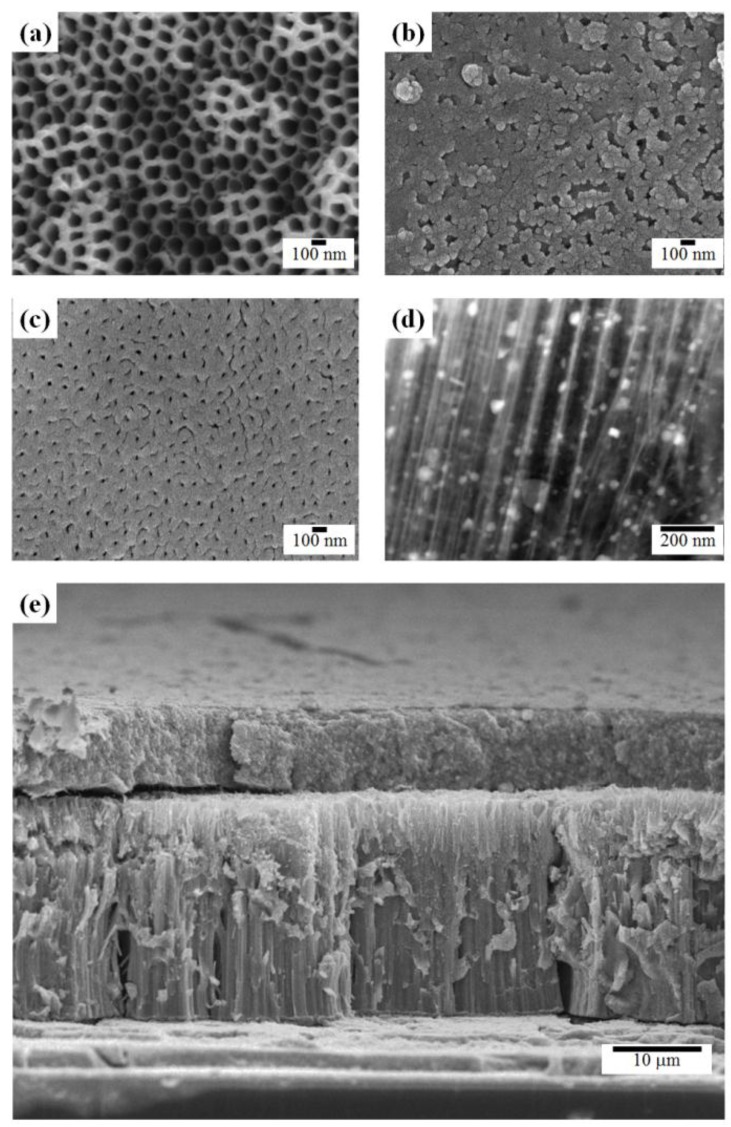
Field emission scanning electron microscope (FE-SEM) images of the (**a**) top, (**b**) bottom, and (**c**) bottom of post–ion milling freestanding TiO_2_ nanotube arrays; (**d**) a high-angle annular dark-field (HAADF) image of Ag NPs in the channel of TiO_2_ nanotube arrays; and (**e**) a side view of the active layer with freestanding TiO_2_ nanotube arrays and a scattering layer.

Field emission scanning electron microscope (FE-SEM) images of freestanding TiO_2_ nanotube (TNT) arrays are shown in Figure 2. The top side of the freestanding TiO_2_ nanotube arrays had 100-nm-diameter pores, as shown in Figure 2a. The bottom layer of closed-ended freestanding TiO_2_ nanotube arrays before ion milling lacked pores, as shown in Figure 2b. However, after ion milling, 20-nm-diameter pores were evident on the bottom layer of open-ended freestanding TiO_2_ nanotube arrays, as shown in Figure 2c. Open-ended TNT arrays can be prepared by chemical etching [35] or physical etching [33,36]. In the chemical etching method, the bottom layers of TNT arrays were easily removed by the etchant. However, the surface morphology and length of TNT arrays were also dissolved in etchant and TNT arrays are fragile when they are attached to a substrate because of their amorphous crystallinity. In the physical etching method, the bottom layer of TNT arrays was removed by the plasma or ion milling process, which is not simple. However, the surface morphology and length of TNT arrays are not damaged in the process and they are very stable when they are attached to a substrate because TNT arrays have the ability to change crystallinity from the amorphous to the anatase phase. After UV irradiation using a silver source, ~30 nm Ag NPs were seen in the channels of freestanding TiO_2_ nanotubes in high-angle annular dark-field (HAADF) images, as shown in Figure 2d. The length of the TiO_2_ nanotubes was ~22 µm and the length of the scattering layer, which consisted of 400 nm TiO_2_ NPs, was ~10 µm.

**Figure 3:**
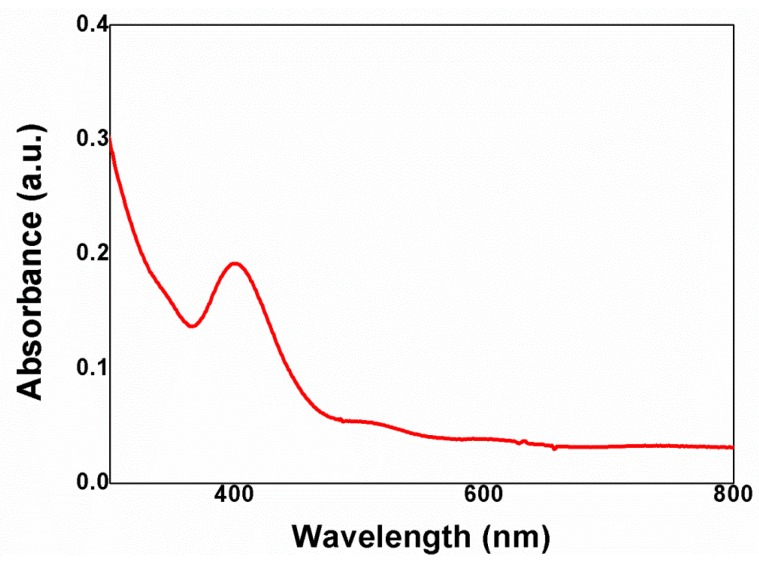
Ultraviolet-visible (UV-vis) spectrum of Ag NP-functionalized TiO_2_ nanotubes.

The ultraviolet-visible (UV-vis) spectrum of Ag NPs in the channels of freestanding TiO_2_ nanotubes is shown in Figure 3. A broad absorption peak centered at 402 nm was observed. The value is different from what it would be in the general solution phase, which is a 420 nm UV absorbance from 30 nm Ag NPs. This discrepancy may stem from different synthesis and measurement conditions used in this study [37,38,39]; the Ag NPs were synthesized by UV irradiation (at 254 nm) without adding a stabilizer and the Ag NPs were measured under dry conditions. The absorption band of Ag NPs is matched with the dye. cis-diisothiocyanato-bis(2,2’-bipyridyl-4,4’-dicarboxylato) ruthenium(II) bis(tetrabutylammonium) (N719) has two visible absorption bands, 390 nm and 531 nm, [40] that were affected by the plasmon band. Moreover, the shell of Ag NPs was prepared with TiCl_4_ to prevent the trapping of electrons by Ag NPs and to enable better electron transport in the channel of the TiO_2_ nanotube arrays.

### 2.3. DSSCs with Closed-Ended Freestanding TiO_2_ Nanotube Arrays with Channels Containing Ag NPs and Large TiO_2_ NPs

**Figure 4:**
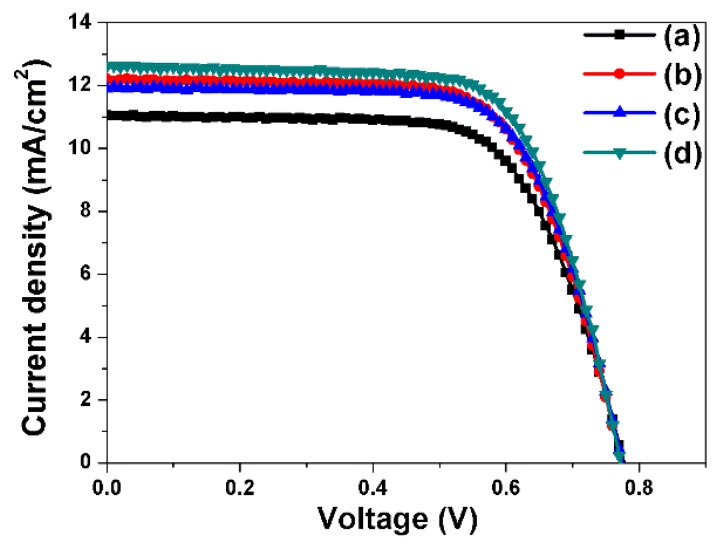
I–V curves of DSSC-based closed-ended freestanding TiO_2_ nanotube arrays fabricated without NPs (**a**), with Ag NPs (**b**), with large TiO_2_ NPs (**c**), and with Ag NPs and large TiO_2_ NPs (**d**).

The photocurrent-voltage curves of DSSCs fabricated using closed-ended freestanding TiO_2_ nanotube arrays measured under air mass 1.5 illumination (100 mW/cm^2^) are shown in Figure 4 and Table 1. Four types of closed-ended freestanding TiO_2_ nanotube array–based DSSCs were fabricated in order to assess the effect of each component on the energy conversion efficiency: closed-ended freestanding TiO_2_ nanotube array–based DSSCs without Ag or large TiO_2_ NPs (a), with Ag NPs (b), with large TiO_2_ NPs (c), and with both Ag NPs and large TiO_2_ NPs (d). The open-circuit voltage (*V_oc_*), short-circuit current (*J_sc_*), fill factor (*ff*), and energy conversion efficiency (η) values are shown in Table 1. The energy conversion efficiency of DSSCs based on closed-ended freestanding TiO_2_ nanotube arrays lacking NPs was 5.86%. The energy conversion efficiencies of DSSCs based on closed-ended freestanding TiO_2_ nanotube arrays with Ag NPs, with large TiO_2_ NPs, and with both Ag NPs and large TiO_2_ NPs were 6.40%, 6.24%, and 6.71%, respectively. The introduction of Ag NPs increased the energy conversion efficiency significantly, by 9.21% (actual efficiency change, from 5.86% to 6.40%) compared to closed-ended freestanding TiO_2_ nanotube array–based DSSCs without Ag and large TiO_2_ NPs, because of increased light harvesting by the plasmonic effect. The introduction of large TiO_2_ NPs also increased the energy conversion efficiency significantly, by 6.48% (actual efficiency, from 5.86% to 6.24%), owing to increased light harvesting by the scattering effect. Moreover, the introduction of both Ag NPs and large TiO_2_ NPs increased the energy conversion efficiency significantly, by 14.50% (actual efficiency, from 5.86% to 6.71%), because of increased light harvesting resulting from both the plasmonic and scattering effects.

### 2.4. DSSCs with Open-Ended Freestanding TiO_2_ Nanotube Arrays with Channels Containing Ag NPs and Large TiO_2_ NPs

**Figure 5:**
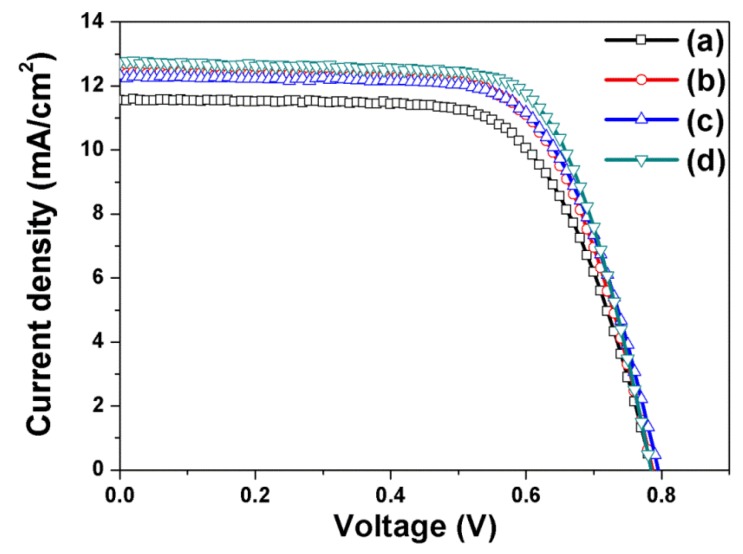
I–V curves of DSSCs based on open-ended freestanding TiO_2_ nanotube arrays fabricated without NPs (**a**), with Ag NPs (**b**), with large TiO_2_ NPs (**c**), and with both Ag NPs and large TiO_2_ NPs (**d**).

The photocurrent-voltage curves of DSSCs fabricated using open-ended freestanding TiO_2_ nanotube arrays are shown in Figure 5 and Table 2; they are useful in assessing the effect of each component on the energy conversion efficiency. Four types of DSSCs based on open-ended freestanding TiO_2_ nanotube arrays were fabricated: open-ended freestanding TiO_2_ nanotube array–based DSSCs without Ag or large TiO_2_ NPs (a), with Ag NPs (b), with large TiO_2_ NPs (c), and with both Ag NPs and large TiO_2_ NPs (d). The *V_oc_*, *J_sc_*, *ff*, and η values are summarized in Table 2. The energy conversion efficiency of DSSCs based on open-ended freestanding TiO_2_ nanotube arrays lacking NPs was 6.12%. The energy conversion efficiencies of DSSCs based on open-ended freestanding TiO_2_ nanotube arrays with Ag NPs, with large TiO_2_ NPs, and with both Ag NPs and large TiO_2_ NPs were 6.68%, 6.62%, and 7.05%, respectively. The introduction of Ag NPs, large TiO_2_ NPs, and both increased the energy conversion efficiency by 9.15%, 8.17%, and 15.20%, respectively. Compared to closed-ended freestanding TiO_2_ nanotube arrays, the energy conversion efficiency of DSSCs based on open-ended freestanding TiO_2_ nanotube arrays was 5.07% (6.71%–7.05%) higher due to enhanced electron transport and electrolyte diffusion [33].

Although TiO_2_ nanotube array-based DSSCs have great potential, as far as we know, the theoretical maximum improvement by Ag NPs or a TiO_2_ scattering layer of TiO_2_ nanotube-based DSSCs has not yet been reported. However, the open-ended TiO_2_ nanotube-based devices exhibited an increase in one-sun efficiency from 5.3% to 9.1%, corresponding to a 70% increase, which is a much higher increase than we achieved [35]. We believe that there is ample room to improve efficiency by combining each parameter in an optimal condition based on theoretical studies.

## 3. Materials and Methods

### 3.1. Materials

Titanium plates (99.7% purity, 0.25 mm thickness, Alfa Aesar, Ward Hill, MA, USA), ammonium fluoride (NH_4_F, Showa Chemical Industry Co., Beijing, China, 97.0%), ethylene glycol (Daejung Chemical, Siheung, Korea, 99%), hydrogen peroxide (H_2_O_2_, Daejung Chemical, Siheung, Korea, 30%), fluorine-doped tin oxide (FTO) glass (Pilkington, St. Helens, UK, TEC-A7), titanium diisopropoxide bis(acetylacetonate) solution (Aldrich, St. Louis, MS, USA, 75 wt % in isopropanol), n-butanol (Daejung Chemical, Siheung, Korea, 99%), TiO_2_ paste (Ti-Nanoxide T/SP, Solaronix, Aubonne, Switzerland), scattering TiO_2_ paste (18NR-AO, Dyesol, Queanbeyan, Australia), silver nitrate (AgNO_3_, Aldrich, St. Louis, MS, USA, 99%), titanium chloride (TiCl_4_, Aldrich, St. Louis, MS, USA, 0.09 M in 20% HCl), dye (cis-diisothiocyanato-bis(2,2’-bipyridyl-4,4’-dicarboxylato) ruthenium(II) bis(tetrabutylammonium) (N719, Solaronix, Aubonne, Switzerland), chloroplatinic acid hexahydrate (H_2_PtCl_6_·6H_2_O, Aldrich, St. Louis, MS, USA), 1-butyl-3-methyl-imidazolium iodide (BMII, Aldrich, St. Louis, MS, USA, 99%), iodine (I_2_, Aldrich, St. Louis, MS, USA, 99%), guanidium thiocyanate (GSCN, Aldrich, St. Louis, MS, USA, 99%), 4-tertbutylpyridine (TBP, Aldrich, St. Louis, MS, USA, 96%), acetonitrile (CH_3_CN, Aldrich, St. Louis, MS, USA, 99.8%), and valeronitrile (CH_3_(CH_2_)_3_CN, Aldrich, St. Louis, MS, USA, 99.5%) were obtained from commercial manufacturers.

### 3.2. Preparation of Freestanding TiO_2_ Nanotube Arrays

TiO_2_ nanotube arrays were prepared by anodization of thin Ti plates. Ti anodization was carried out in an electrolyte composed of 0.8 wt % NH_4_F and 2 vol % H_2_O in ethylene glycol at 25 °C and at a constant voltage of 60 V direct current (DC) for 2 h. After being anodized, the Ti plates were annealed at 500 °C for 1 h under ambient conditions to improve the crystallinity of the TiO_2_ nanotube arrays, and then a secondary anodization was conducted at a constant voltage of 30 V DC for 10 min. The Ti plates were immersed in 10% H_2_O_2_ solution for several hours in order to detach the TiO_2_ nanotube arrays from the Ti plates and produce freestanding TiO_2_ nanotube arrays. The bottom of the TiO_2_ nanotube arrays was removed by ion milling with Ar^+^ bombardment for several minutes in order to prepare open-ended freestanding TiO_2_ nanotube arrays.

### 3.3. Preparation of Ag NPs in the Channel of Freestanding TiO_2_ Nanotube Arrays

A TiO_2_ blocking layer was formed on FTO glass by spin-coating with 5 wt % titanium di-isopropoxide bis(acetylacetonate) in butanol, followed by annealing at 500 °C for 1 h under ambient conditions to induce crystallinity. A TiO_2_ paste was coated onto the TiO_2_ blocking/FTO glass using the doctor blade method, and closed- and open-ended freestanding TiO_2_ nanotube arrays were then introduced onto the TiO_2_ paste. The substrate was annealed at 500 °C for 1 h under ambient conditions to enhance the adhesion between the TiO_2_ NPs and freestanding TiO_2_ nanotube arrays. The substrate was dipped in 0.3 mM AgNO_3_ aqueous solution and exposed to 254 nm UV irradiation. The substrate was treated with 10 mM TiCl_4_ solution at 50 °C for 30 min and then annealed at 500 °C for 1 h.

### 3.4. Fabrication of DSSCs with Freestanding TiO_2_ Nanotube Arrays with Channels Containing Ag NPs

A substrate that consisted of freestanding TiO_2_ nanotube arrays with channels containing Ag NPs was coated with ~400 nm TiO_2_ NPs for scattering and then annealed at 500 °C for 1 h under ambient conditions to induce crystallinity and adhesion. The substrate was immersed in a dye solution at 50 °C for 8 h to function as a working electrode. The working electrode was sandwiched with a counter electrode, Pt on FTO glass, by a 60-μm-thick hot-melt sheet and filled with electrolyte solution composed of 0.7 M 1-butyl-3-methyl-imidazolium iodide (BMII), 0.03 M I_2_, 0.1 M guanidium thiocyanate (GSCN), and 0.5 M 4-tertbutylpyridine (TBP) in a mixture of acetonitrile and valeronitrile (85:15, *v*/*v*).

### 3.5. Instruments

The morphology, thickness, size, and presence of Ag NPs in the channels of freestanding TiO_2_ nanotube arrays were confirmed using a field emission scanning electron microscope (FE-SEM, JSM-6330F, JEOL Inc., Tokyo, Japan) and the high angular annular dark field (HAADF) technique with a scanning transmission electron microscope (TEM, JEM-2200FS, JEOL Inc., Tokyo, Japan). The current density−voltage (*J*−*V*) characteristics and the incident photon-to-current conversion efficiency (IPCE) of the DSSCs were measured using an electrometer (Keithley 2400, Keithley Instruments, Inc., Cleveland, OH, USA) under AM 1.5 illumination (100 mW/cm^2^) provided by a solar simulator (1 kW xenon with AM 1.5 filter, PEC-L01, Peccell Technologies, Inc., Yokohama, Kanagawa, Japan), or using a solar cell IPCE measurement System(K3100, McScience Inc., Suwon, Korea) with reference to the calibrated diode.

## 4. Conclusions

In this study, we compared the natural consequences of altering three parameters, the plasmonic effect, the scattering effect, and open- *vs.* closed-ended freestanding TiO_2_ nanotubes, as a basic means of exploring improvements in efficiency. We demonstrated that the plasmonic and scattering effects enhanced the energy conversion efficiency of freestanding TiO_2_ nanotube arrays in DSSCs. Ag NPs were added to the channels of TiO_2_ nanotube arrays by UV irradiation to induce a plasmonic effect, and large TiO_2_ NPs were introduced to TiO_2_ nanotube arrays to induce a scattering effect. The energy conversion efficiency of DSSCs with both Ag NPs and large TiO_2_ NPs was higher than that of DSSCs without Ag NPs owing to the plasmonic effect, and it was higher than that of DSSCs without large TiO_2_ NPs owing to the scattering effect. Compared to closed-ended freestanding TiO_2_ nanotube arrays, open-ended freestanding TiO_2_ nanotube arrays [40] exhibited enhanced energy conversion efficiency. We demonstrate that Ag NPs, TiO_2_ NPs, and open-ended freestanding TiO_2_ nanotube arrays enhanced the energy conversion efficiency; furthermore, the combination of all components exhibited the highest energy conversion efficiency. Our research suggests that the energy conversion efficiency of DSSCs is improved by both the plasmonic and scattering effects. This knowledge has applications in organic solar cells, hybrid solar cells, and perovskite solar cells.

## Figures and Tables

**Table 1:** Photovoltaic properties of dye-sensitized solar cells (DSSCs) based on closed-ended freestanding TiO_2_ nanotube arrays.

DSSCs	*J_sc_* (mA/cm^2^)	*V_oc_* (V)	*ff*	η (%)
(a) Closed-ended freestanding TiO_2_ nanotube arrays without any NPs	11.05	0.78	0.68	5.86
(b) Closed-ended freestanding TiO_2_ nanotube arrays with Ag NPs	12.22	0.77	0.68	6.40
(c) Closed-ended freestanding TiO_2_ nanotube arrays with large TiO_2_ NPs	11.90	0.76	0.69	6.24
(d) Closed-ended freestanding TiO_2_ nanotube arrays with Ag NPs and large TiO_2_ NPs	12.63	0.77	0.69	6.71

**Table 2:** Photovoltaic properties of DSSCs based on open-ended freestanding TiO_2_ nanotube arrays.

ADSSCs	*J_sc_* (mA/cm^2^)	*V_oc_* (V)	*ff*	η (%)
(a) Open-ended freestanding TiO_2_ nanotube arrays without any NPs	11.56	0.79	0.67	6.12
(b) Open-ended freestanding TiO_2_ nanotube arrays with Ag NPs	12.45	0.79	0.68	6.68
(c) Open-ended freestanding TiO_2_ nanotube arrays with large TiO_2_ NPs	12.33	0.79	0.68	6.62
(d) Open-ended freestanding TiO_2_ nanotube arrays with Ag NPs and large TiO_2_ NPs	12.74	0.78	0.71	7.05
